# Dynamic Neural Network of Insight: A Functional Magnetic Resonance Imaging Study on Solving Chinese ‘Chengyu’ Riddles

**DOI:** 10.1371/journal.pone.0059351

**Published:** 2013-03-15

**Authors:** Qingbai Zhao, Zhijin Zhou, Haibo Xu, Shi Chen, Fang Xu, Wenliang Fan, Lei Han

**Affiliations:** 1 Key Laboratory of Adolescent Cyberpsychology and Behavior (CCNU), Ministry of Education, Wuhan, People’s Republic of China; 2 Key Laboratory of Human Development and Mental Health of Hubei Province, School of Psychology, Central China Normal University, Wuhan, People’s Republic of China; 3 MRI Center of Union Hospital, Huazhong University of Science and Technology, Wuhan, People’s Republic of China; 4 School of Psychology, Shandong Normal University, Jinan, People’s Republic of China; Catholic University of Sacro Cuore, Italy

## Abstract

The key components of insight include breaking mental sets and forming the novel, task-related associations. The majority of researchers have agreed that the anterior cingulate cortex may mediate processes of breaking one’s mental set, while the exact neural correlates of forming novel associations are still debatable. In the present study, we used a paradigm of answer selection to explore brain activations of insight by using event-related functional magnetic resonance imaging during solving Chinese ‘chengyu’ (in Chinese pinyin) riddles. Based on the participant’s choice, the trials were classified into the insight and non-insight conditions. Both stimulus-locked and response-locked analyses are conducted to detect the neural activity corresponding to the early and late periods of insight solution, respectively. Our data indicate that the early period of insight solution shows more activation in the middle temporal gyrus, the middle frontal gyrus and the anterior cingulate cortex. These activities might be associated to the extensive semantic processing, as well as detecting and resolving cognitive conflicts. In contrast, the late period of insight solution produced increased activities in the hippocampus and the amygdala, possibly reflecting the forming of novel association and the concomitant “Aha” feeling. Our study supports the key role of hippocampus in forming novel associations, and indicates a dynamic neural network during insight solution.

## Introduction

Since Köhler observed that chimpanzees could resolve problems suddenly rather than by an approach of trial and error [1], the processing of insight has attracted attention of many researchers. Following the early Gestalt psychologists, who thought that insightful problem-solving was based on a reconstruction to the whole problem, some cognitive psychologists proposed that it is due to the unsuitable representations of problem that led to the failure of effective problem solving in many situations. They further suggested that the representation change such as constraint relaxation and chunk decomposition should be the crucial process of insight [2]–[4]. This theory was successful in interpreting visual-representation-based insightful problem solving, such as nine-dot problem.

With the development of neuroimaging technique, especially from 1990s onwards, the investigations on the neural correlates of insight flourished [5]–[10]. However, since a number of homogenous mental events which can be repeatedly observed are required for the neuroimaging approach, the classic insightful paradigms such as nine-dot problem and six-matchstick problem are no longer suitable [11]. Thus, a variety of insightful paradigms by using semantic representation have been applied, such as riddles and compound remote associates (CRA) problems [5], [6], [8]. The generation-selection model [12] has successfully interpreted the cognitive processing of these problems. According to this theory, the first step of insightful problem solving is to activate a variety of information and to form the temporary connection between them. The second step is to choose the task-related connection to solve the problem successfully. The key components of this model include processes to break the mental sets and to form novel, task-related associations among the old nodes of concepts or cognitive skills [6], [13].

Luo and Niki [6] used fMRI to record neural activities during insight by providing a trigger (the solution) to catalyze insightful solving processes to Japanese riddles. They found that the hippocampus played an important role in both breaking mental set and forming novel associations. Other studies demonstrated the availability of the paradigm to catalyze insight in studying breaking the mental sets, and showed that the anterior cingulate cortex (ACC) was the key brain region associated to this process [14]–[16].

However, providing a trigger made the problem solving more an apperception rather than an insight in the strict sense. Therefore, a learning-testing paradigm was introduced into Chinese logogriphs by Qiu et al. to explore the brain mechanism of insightful problem solving. The event-related potential (ERP) studies indicated that the ACC mediated the cognitive conflicts in both stages of mental preparatory and problem solving [17], [18]. And other fMRI results showed that the insight activated more in the precuneus, the left inferior/middle frontal gyrus, the inferior occipital gyrus and the cerebellum. These activities might be linked with successful prototype events retrieval, forming novel association and breaking mental sets, and re-arrangement of visual stimulus [5].

The CRA problems were also applied to investigate insight. In such a paradigm, insight and noninsight conditions were distinguished based on subsequent responses of participants’ subjective feeling [8], [9], [19]. Both fMRI and electroencephalogram (EEG) found an increased activity at the right anterior superior temporal gyrus during insight. This brain area was thought to be associated with making connections across distantly related information during comprehension [8]. These studies supported the right-brain dominance theory of insight.

Besides, Aziz-Zadeh et al. [20] used the anagrams to study insight, and found verbal insight solutions activated a distributed neural network, including the bilateral insula, the right prefrontal cortex and the ACC. However, in “Chinese anagrams”, the fusiform and the right superior temporal gyri were found to be involved in insight solution [21].

As reviewed above, the majority of studies agreed that the ACC mediates processes of breaking one’s mental set, while the exact neural correlates of forming novel associations are still debatable. In our opinion, the different tasks/experimental paradigms, as well as different definitions of timing insight event, may be the main cause leading to the disagreement.

In the learning-testing paradigm, the problem solving should be similar to the analogy thinking in some sense. The learning of prototype events reduces the difficulty of problem solving so that participants can find a solution on their own initiative. However, the learning of prototype events also weakens the insight effect. At the same time, the forming of novel associations should occur at the end of insightful problem solving, while the insight events defined by Qiu [5] were time-locked at the beginning of the presentations of target logogriphs. This stimulus-locked analysis would catch the process of breaking mental sets, but not forming novel associations. Therefore, it seems that the left lateral prefrontal cortex should not be the key brain region for forming novel associations.

In the CRA problems, the mean reaction time of producing insight solutions was about 10 seconds. Jung-Beeman et al. [8] marked the insight events at the point about 2 seconds prior to each solution button pressing. This response-locked analyis could catch the process of forming associations. However, since the CRA problems are hybrid insight problems which can be solved through either insight or noninsight processes [22], [23], the associations formed during CRAs might not be always novel. Moreover, as a well-known brain region linked with language ability, the right superior temporal gyrus was activated in processing the literal meaning of idioms [24]. In that sense, the right superior temporal gyrus might be associated with the activation of extensive semantic network.

In the paradigm of providing a trigger to catalyze insight [6], the mean reaction time of insight solving was less than the repetition time of fMRI scanning (about 2 seconds). Thus, the definition of timing insight events (as the beginning of the presentations of the trigger) might catch both the breaking of mental sets and the forming of novel associations. However, the short reaction times led to the fact that the different periods of insight solving could not be separated by fMRI data analysis. Additionally, the activation of the hippocampus was not found in another research by using similar paradigm [14], though it might be associated with forming novel associations due to its function in path reorientation [25] and relational memory [26].

In the present study, we adopted Chinese ‘chengyu’ (in Chinese pinyin) riddles to explore the underlying neural mechanism of insight. ‘Chengyu’ is a type of traditional Chinese idiomatic expressions. Most of them consist of four characters. As most ‘chengyu’ are often intimately linked with the myth or historical event from which they were derived, their meaning usually surpasses the simple combination of the four characters. Different from the English idioms, which have the literal and figurative meanings [27], these ‘chengyu’ only have the figurative meaning and their literal meanings are unintelligible. It is worth to mention that there are some other ‘chengyu’ that are not born of a well-known fable. This type of ‘chengyu’ is just the shortened expression of its literal meaning, and they have no figurative meanings.

As each ‘chengyu’ only has one meaning, the meanings of its four component characters are constrained by the chunk of the ‘chengyu’. This produces a mental set, which prevents the successful riddle solving because the riddles aim at an unconstrained meaning of the key character rather than the meaning of the ‘chengyu’ as a whole. To solve the riddle, the chunk of ‘chengyu’ must be decomposed, and extensive meanings of individual characters must be explored and retrieved. For example, the answer of the riddle ‘shan zhan er duo mou’ (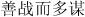, means adept at fighting and planning) is the chengyu ‘jing da xi suan’ (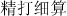, means being very careful in reckoning). The key character in this riddle is ‘da’ (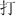, one of its meanings is to hit), corresponding to ‘zhan’ (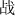, with the meaning to fight). However, inside the ‘chengyu’, the ‘da’ (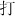) is bound with ‘suan’ (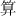). And the meaning of their combination ‘da suan’ (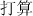) is to plan or to reckon. Obviously, the successful riddle solving is relied on the successful constraint relaxation to the key character or the successful chunk decomposition. This is theoretically similar with the visual chunk decomposition of Chinese characters [4]. Once extensive meanings of key character were retrieved, a number of temporary connections between the riddle and the ‘chengyu’ would be formed, and then the riddle would be solved by the selection process of task-related connections.

To explore the neural correlates of forming novel associations, another ‘chengyu’ that is similar in meaning to the riddle is used as the control condition (see in Methods). This ‘chengyu’ is normally associated with the riddle. For example, the ‘chengyu’ normally associated with the riddle ‘shan zhan er duo mou’ (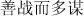, means adept at fighting and planning) is “zu zhi duo mou” (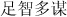, means being able and adept at planning). Thus, by directly manipulating the novelty of associations between the riddle and answer, we can accurately detect the neural correlates of forming novel associations.

Additionally, since different analyze methods, such as stimulus-locked or response-locked approaches, may be a factor resulting in the inconsistent findings in previous studies, both approaches were adopted in this study to detect the neural activity of early and late periods of insight solution respectively. Although fMRI is usually linked with poor temporal resolution, the timing of different periods of insight can be resolved by using the temporal analysis, in which the brain regions related to different periods have different peaks of the time course. Note that the separation of different periods needs a longer solution time, maybe double of repetition time of scanning or even more.

## Methods

### Participants

As paid volunteers, 20 undergraduates or graduates (13 women, 7 men), aged 21–35 years (mean age, 23.6 years) from Central China Normal University (CCNU), participated in the experiment, and gave their informed consent according to the requirements of Institutional Review Board of CCNU. All participants were healthy, right-handed, and had normal or corrected to normal vision. Two participants were excluded from analysis due to their experiencing of less than 15% normal associations during the experiment. Another participant was excluded due to the excessive head motion during fMRI scanning.

### Stimuli and Task

A Chinese ‘chengyu’ riddle may be a phrase, or a saying, and its answer is a four-character ‘chengyu’. Since there is a process of representation change when the participants tried to associate the riddle with the original answer, it is considered as the answer with novel association. In the current work, a control with normal association was produced in a pretest. A group of subjects were asked to report the four-character ‘chengyu’ that came to mind first when they saw the riddle in the pretest. Mostly, they could not find the novel answer and gave some different answers. The ‘chengyu’ with the highest frequency was chosen as the control. Thus, there are two answers, one of which is novel, and the other is normal. Taking the example used in the Introduction, the novel answer to the riddle ‘shan zhan er duo mou’ (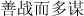, means adept at fighting and planning) is the ‘chengyu’ of ‘jing da xi suan’ (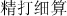, means being very careful in reckoning), while its normal answer is ‘zu zhi duo mou’ (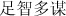, means being able and adept at planning).

In order to determine the difference between the answers with novel and normal associations, we had another group of subjects (totally 32) to rate their understanding of the Reasonability (matching with the answers to riddles) and Novelty on a scale of 1 to 5 for each of the 120 riddles. In the end, 84 riddles whose answers (both novel and normal ones) were evaluated as reasonable (mean scores > 3.5) were selected as the test riddles. Results showed that there was a significant difference in novelty [paired t-test, t (83)  = 16.84, p<0.001] between the answers with novel (mean score  = 3.6) and normal association (mean score = 2.6).

**Figure 1:**
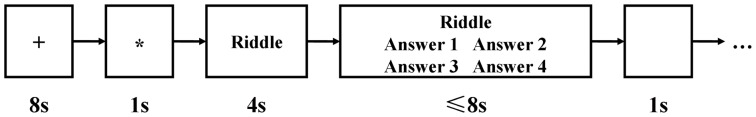
The flow map of the formal experiment.

To familiarize the participants with the procedure and pace of this task, participants were trained with another set of 10 similar materials before they were put into the scanner. In the formal experiment, 84 test riddles were presented one by one with an event-related design. There was not any repetition of stimuli in the test. The Chinese characters, appearing in both the riddles and answers, had a font size of 28 (Song Ti font). The experimental paradigm was illustrated in Figure 1. The trial began with an 8-second black plus, a sign for rest, and a star sign for 1 second, followed by a warning of the presentation of riddle. After the riddle was displayed for 4 seconds, the novel association answer, normal association answer and two answers with no associations were presented. Participants were asked to select a novel and reasonable answer among these options within a limited 8-second period. Then was a 1-second blank followed by the next trial. The spatial positions were balanced among the different answers.

Since it is difficult to produce the novel answer on subjects’ own initiative , while it seems an apperception if only providing the novel answer, the answer selection paradigm used here is an inevitable compromise [28]. According to the selections of participants, the trials are classified into insight and noninsight solutions, respectively. Because including a process of representation change, the selection of novel answer is indeed an insight-based solution; however, as the selection of normal answer means the representation is not changed successfully, it can be considered as noninsight-based solution. Although it might increase subjects’ suppression, the simultaneous presentation of the novel and normal answers is still necessary in the answer selection paradigm. If only the novel or normal answer was presented with unrelated ones, subjects would get the target easily. That would be similar with the paradigm of providing the trigger [6]. Additionally, subjects’ suppression exists in both insight and noninsight solutions, and it can be eliminated by the comparison in the stage of data analysis.

### fMRI Acquirement

During MRI scanning, whole brain T2*-weighted echo planar imaging, based on blood oxygenation level-dependent contrast (EPI-BOLD) fMRI data, was acquired with a Siemens Trio 3.0-T MR-scanner using a standard head coil at the MRI Center of Wuhan Union Hospital. 32 interleaved slices, covering the entire brain, were acquired using a gradient-echo echo-planar pulse sequence. The slice thickness was 3.75 mm and the voxel size was 3 mm × 3 mm (TR  =  2 s, TE  =  30 ms, FA = 78°,FOV = 192×192 mm, Matrix size  =  64×64). Head motion was restricted with plastic braces and foam padding. The whole scanning sequence was divided into 2 runs, each consisted of 42 trials.

### fMRI Data Analysis

The statistical parametric mapping (SPM5, http://www.fil.ion.ucl.ac.uk/spm/) was used for image preprocessing and voxel-based statistical analysis. Scans were first slice-time corrected, realigned, normalized (using the functional EPI template provided in SPM2), and smoothed (a Gaussian kernel with a full width at the full width at half maximum – FWHM of 8 mm). The resultant images had cubic voxels of 2 × 2 × 2 mm.

Two types of events, the insight solution (IS) and the noninsight solution (NS), were defined according to participants’ selections to the answers. For each type, both stimulus-locked and response-locked analyses were conducted. In the stimulus-locked analysis, the event was defined as the presentation of the answers which was time-locked to the onset of the answer’s presentation with zero duration. This event was considered as the early period activity of problem solving (EIS or ENS). Then, the time vector of the event was convoluted by the classic haemodynamic response function (HRF). Finally, by the general linear model, the activated brain regions associated with the EIS and ENS, as well as the differences between the two conditions, were obtained for each participant, and then combined in a random effect analysis to identify differences consistent across all participants. The thresholds were at p<0.001 (uncorrected for multiple comparisons) and 50 or more contiguous voxels (more than 10 original-sized voxels) for insight versus noninsight. The response-locked analysis was similar to the stimulus-locked analysis, except that the event was time-locked to 2 seconds prior to solution button pressing with zero duration. Similarly, this event was considered as the late period activity of problem solving (LIS or LNS).

## Results

### Behavioral Performance

On average, in 61.7% of trials participants selected the answers with novel associations (average RT was 3.70 s with a standard deviation of 0.78 s), and in 26.3% of trials they selected the answers with normal associations (average RT was 4.06 s with a standard deviation of 0.94 s). There was a significant difference between the reaction times of the two conditions [paired t-test, t (16)  =  -5.63, p<0.001]. The larger trial percentage and short reaction time of insight solutions might result from the instruction before the experiment that asked participants to select a novel and reasonable answer.

### fMRI Results

#### Insight versus Noninsight in the Early Period of Solution

This contrast (EIS-ENS) examined brain areas that were more activated in the early period of insight solutions relative to the noninsight solutions. Several peaks of activation were found, including the left superior temporal pole, inferior temporal gyrus, anterior cingulate cortex and middle frontal gyrus, the right inferior frontal gyrus and parahippocampal gyrus, and the bilateral angular gyrus and middle temporal gyrus (see in Table 1). There were no voxels that were significantly more active for the early processing of noninsight solutions than the insight solutions.

**Table 1:** Brain areas more activated in insight than in noninsight solution.

Area	BA	Voxels	*x*	*y*	*z*	T	Z
***Insight > Noninsight in the early period of solution***							
Left superior temporal pole	38	752	–*28*	*10*	–*28*	6.31	4.41
Left inferior temporal gyrus	21		–*42*	*0*	–*38*	5.95	4.26
Left anterior cingulate cortex	32	58	–*10*	*42*	*8*	6.06	4.31
Left middle frontal gyrus	8	148	–*44*	*20*	*48*	5.96	4.27
Right angular gyrus	40	326	*50*	–*56*	*36*	5.81	4.20
Right middle temporal gyrus	21	126	*54*	*2*	–*30*	5.50	4.07
Left angular gyrus	39	1070	–*50*	–*56*	*36*	5.81	4.20
Left middle temporal gyrus	21		–*52*	–*30*	–*2*	5.04	3.84
Right middle temporal gyrus	21	248	*60*	–*48*	*8*	4.94	3.79
Right inferior frontal gyrus	47	81	*24*	*14*	–*20*	4.74	3.69
Right parahippocampal gyrus	28		*24*	*4*	–*32*	4.49	3.56
***Insight > Noninsight in the late period of solution***							
Right middle temporal gyrus	21	834	*68*	–*44*	–*6*	7.46	4.83
Right middle temporal gyrus	37		*56*	–*56*	*8*	5.52	4.07
Left olfactory	34	1720	–*24*	*6*	–*18*	7.02	4.68
Left middle temporal gyrus	20		–*54*	–*8*	–*26*	6.73	4.57
Left hippocampus	20		–*26*	–*10*	–*20*	6.20	4.36
Right angular gyrus	39	158	*46*	–*56*	*36*	6.88	4.63
Right putamen	48	349	*22*	*6*	–*12*	6.50	4.48
Right amygdala	34		*28*	–*4*	–*10*	5.96	4.27
Right hippocampus	20		*30*	–*6*	–*20*	4.37	3.49
Left middle frontal gyrus	46	124	–*38*	*22*	*42*	6.38	4.44
Left anterior cingulate cortex	10	1032	–*4*	*52*	*2*	5.83	4.21
Left medial frontal gyrus	10		–*8*	*54*	*18*	5.79	4.19
Left middle temporal gyrus	21	333	–*52*	–*32*	–*2*	5.49	4.06
Left angular gyrus	39	642	–*52*	–*64*	*40*	4.80	3.73
Left inferior parietal gyrus	40		–*60*	–*48*	*44*	4.78	3.71

BA, Brodmann area. Coordinates (x,y,z) were the MNI (Montreal Neurological Institute) coordinates. The thresholds were set at p<0.001 (uncorrected for multiple comparisons) and 50 or more contiguous voxels. T- and Z-scores of the activations were also shown.

#### Insight versus Noninsight in the Late Period of Solution

This contrast (LIS-LNS) examined brain areas that were more activated in the late period of insight solutions relative to the noninsight solutions. Several peaks of activation were found, including the left olfactory, middle frontal gyrus, anterior cingulate cortex, medial frontal gyrus and inferior parietal gyrus, the right putamen and amygdala, and the bilateral middle temporal gyrus, hippocampus and angular gyrus (see in Table 1). There were no voxels that were significantly more active for the late processing of noninsight solutions than the insight solutions.

#### Timecourse Activity of Regions Associated with Insight

**Figure 2:**
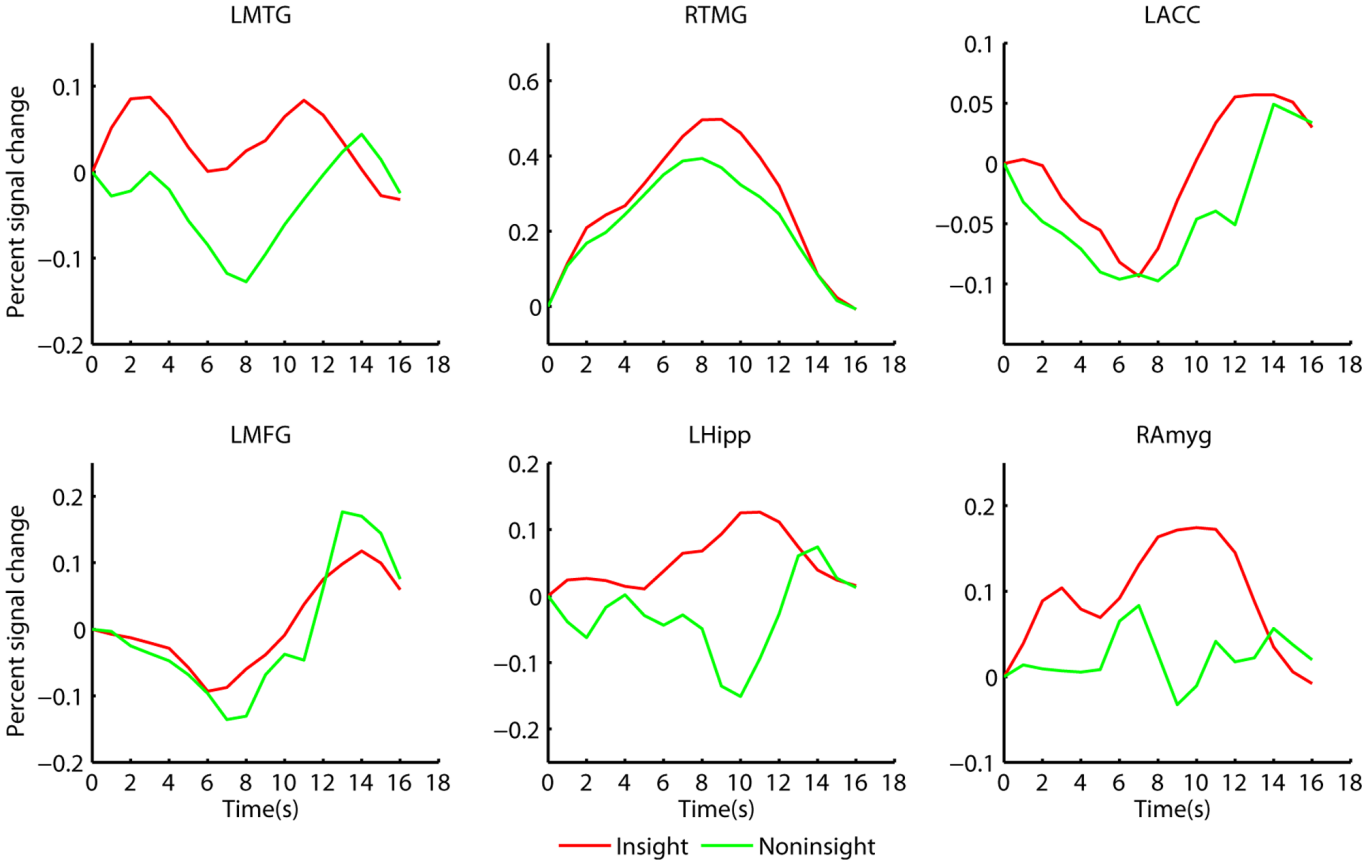
Average signal change of the interested brain regions, following the onset (time = 0) of answer presentation for insight solution (red line) and noninsight solution (blue line). LTMG means left middle temporal gyrus with the peak at (–52,–30,–2) in MNI space, RTMG means right middle temporal gyrus with the peak at (60,–48,8), LACC means left anterior cingulate cortex with the peak at (60,–48,8), LMFG means left middle frontal gyrus with the peak at (–44,20,48), Lhipp means left hippocampus with the peak at (–26,–10,–20) and Ramyg means right amygdala with the peak at (28,–4,–10). Note that the timecourse data were linearly interpolated from the original 2-s resolution down to 1-s resolution to obtain the smoothed curves.

To clarify the differences between the early and late period activity of insight, the timecourse activity of the brain regions associated with insight was visualized. According to the HRF by SPM, the haemodynamic response to the EIS would delay for six seconds from the onset of the answer presentation, and that to the LIS would delay for ten seconds (the delay time plus mean solution time). As shown in Figure 2, the haemodynamic response of the left and right middle temporal gyri, left anterior cingulate cortex and middle frontal gyrus started at the beginning of the answers presentation and continued until the riddle was solved (specifically, the timecourse of left middle temporal gyrus has a bi-peak); while that of the left hippocampus and right amygdala started just before the riddle was solved (about four seconds after the onset of answer presentation), with a peak at ten seconds. These were consistent with the statistical results above.

## Discussion

According to the generation-selection model, the insightful riddle solving meant to activate a variety of information covering the riddle and answers, to decompose the chunk of target ‘chengyu’, and then to form the novel, task-related associations. Accordingly, the process of riddle solving was divided into the early and late periods of solution, which can be separated by the stimulus-locked and response-locked anslyses of fMRI data. In the early period, the insight activated more in broad lateral temporal areas, left middle frontal gyrus and ACC; while in the late period, a broader network of brain areas was activated, including the lateral temporal areas, left middle frontal gyrus, ACC, hippocampus, amygdala. These areas suggested a dynamic neural network of insightful problem solving. Their roles were discussed below.

### Middle Temporal Gyrus in Extensive Semantic Processing

The bilateral middle temporal gyri were activated in both early and late periods of insight solution relative to the noninsight solution. These brain areas are well-known in semantic processing [29], [30].

In the present study, the normal association was formed just when the whole meaning of the normal answer was understood, while the forming of novel association needed to decompose the target ‘chengyu’ and to activate the extensive (normal and novel) meanings of the key character. It was found that the processing of the novel meaning of idioms was associated with increased activity in the right middle and superior temporal gyri [24]. It was proposed that the right superior temporal gyrus is the key brain area in forming connections across distantly related information [8], [31]. The right superior temporal gyrus was not emphasized in other insight studies [32], probably due to its well-established function in language processing. Additionally, it was found by others that the bilateral superior temporal gyrus , rather than only right side one, were activated in insight problem solving [6]. In the present study, it was the bilateral middle temporal gyrus rather than the right superior temporal gyrus that was more activated in insight solution. Consistent with some others’ work [32], the current results provide no support to the right-brain dominance theory in insight.

Although the insightful problem solving comes suddenly, it still needs activations of extensive and detailed semantic information in order to form novel associations. Thus, the increased activity of temporal areas might be associated with the activation of extensive semantic network rather than the novel associations. This was supported by the fact that there was no significant difference in the activation of middle temporal gyrus between the early and late insight effect.

### ACC and Middle Frontal Gyrus in Breaking Mental Sets

The left middle frontal gyrus and ACC were activated in both early and late insight solution relative to the noninsight solution.

Insight means to break the mental sets and to form novel, task-related associations [6],[13]. To break a mental set, subjects need to detect and resolve cognitive conflict [14]. Studies indicated that the anterior cingulate cortex (ACC) was more activated in insight problem solving [14], [15], [18], [20]. Due to its well-known role in cognitive conflict monitoring [33], [34], the ACC was highlighted and proposed to mediate processes of breaking one’s mental set. In addition, it was found that increased activity in ACC, prior to problem presentation, might reflect increased readiness for monitoring of competing responses and for applying of cognitive control mechanisms. The increased ACC activity could also predict subsequent successful solution in term of a sudden insight [17], [19].

After detecting the cognitive conflicts, one should shift its mental sets to solve the conflicts in insight. It was found that the lateral prefrontal cortex, including the inferior and middle frontal gyri, was activated in chunk decomposition of Chinese characters [4], set-shift problems [35] and insightful riddle solving [5], [6], [14]. Due to its role in establishing and shifting the attentional sets [36], [37], the lateral prefrontal cortex was thought to be associated with conflict resolution.

Note that the ACC and middle frontal gyrus were deactivated relative to the resting stage. This was different from some findings [6], [20], but consistent with others [19], in which the activity of the ACC reached the peak during the prepared period but decreased in the middle of the task. It was a reasonable result since the ACC is one part of the default mode network which shows greater activity during resting state than during cognitive tasks [38]–[40].

### Hippocampus in Forming Novel Associations

The bilateral hippocampi were activated in the late (but not the early) period of insight solution compared with the noninsight solution.

In the first neuroimaging study on insight [6], the right hippocampus was found activated in the insight event. Due to its function in path reorientation [25] and relational memory [26], the hippocampus was proposed to play an important role in both of breaking mental set and forming novel associations. As discussed above, the ACC and lateral prefrontal cortex should be the key brain area responsible for processes of breaking one’s mental set. Thus, the hippocampus might be mainly associated with the forming of novel associations. This was supported by two facts. First, an increased activity of hippocampus was detected during encoding of novel visual stimuli [41], and hippocampal damage altered the ERPs in response to novel stimuli [42]. Second, in task to judge the relationship of semantically related or unrelated word pairs, the hippocampus was responsive to the retrieval of semantic associations [43].

Our result supported the role of hippocampus in forming novel associations. Firstly, we manipulated the novelty of association between the riddle and answer, and found an increased activity of hippocampus in the selections to the novel answers. Secondly, the hippocampus showed more activation than the noninsight solution only in the late period of insight solution. It was the time of forming novel associations.

### Amygdala in “Aha” Feeling

The amygdala was activated in the late period of insight solution relative to the noninsight solution.

Studies demonstrated that the amygdala was associated with emotional learning and expression [44]. Although the majority of studies reported the amygdala was sensitive to negative stimuli [45], recent studies revealed that it was also involved in positive emotions [46], [47].

Both the cognitive processing of forming novel associations and the corresponding “Aha” feeling should be included in the current paradigm of insight solution. Due to its role in emotion, the amygdala was proposed to reflect the subjective experience during insight. As it is approaching to the completion of insightful solution, the insight feeling is strengthened, and the activity of amygdala is also increased.

## Conclusion

Our data demonstrated that the middle temporal gyrus, the middle frontal gyrus, the ACC, the hippocampus and the amygdala, are more activated during the insight solution than during the noninsight solution. These brain areas consist of a dynamic neural network, associated with the cognitive and affective functions during insight of Chinese ‘chengyu’ riddles solving. The increased activity of middle temporal gyrus may reflect the activation of extensive semantic network; the activation of lateral prefrontal cortex and ACC may be responsible for detecting and resolving cognitive conflict; the hippocampus seems to be the key brain areas in forming novel associations; and the activation of amygdala might reflect the subjective experience during insight. Although the superior temporal gyrus was proposed previously to be the key brain area in forming connections across distantly related information, this region is not found activated during insight in the present study, and the right-brain dominance theory on insight is thus not supported by our results.
